# Membrane Protein Stability Analyses by Means of Protein Energy Profiles in Case of Nephrogenic Diabetes Insipidus

**DOI:** 10.1155/2012/790281

**Published:** 2012-03-15

**Authors:** Florian Heinke, Dirk Labudde

**Affiliations:** Department of Mathematics, Natural and Computer Sciences, Hochschule Mittweida, University of Applied Sciences, Technikumplatz 17, 09648 Mittweida, Germany

## Abstract

Diabetes insipidus (DI) is a rare endocrine, inheritable disorder with low incidences in an estimated one per 25,000–30,000 live births. This disease is characterized by polyuria and compensatory polydypsia. The diverse underlying causes of DI can be central defects, in which no functional arginine vasopressin (AVP) is released from the pituitary or can be a result of defects in the kidney (nephrogenic DI, NDI). NDI is a disorder in which patients are unable to concentrate their urine despite the presence of AVP. This antidiuretic hormone regulates the process of water reabsorption from the prourine that is formed in the kidney. It binds to its type-2 receptor (V2R) in the kidney induces a cAMP-driven cascade, which leads to the insertion of aquaporin-2 water channels into the apical membrane. Mutations in the genes of V2R and aquaporin-2 often lead to NDI. We investigated a structure model of V2R in its bound and unbound state regarding protein stability using a novel protein energy profile approach. Furthermore, these techniques were applied to the wild-type and selected mutations of aquaporin-2. We show that our results correspond well to experimental water ux analysis, which confirms the applicability of our theoretical approach to equivalent problems.

## 1. Introduction

Membrane proteins play important roles in many biological processes. Although the total number of known membrane protein structures has increased from 337 to 1515 structures within the last eight years, the high degree of redundancy and the average quality of these structures reduce the overall condition of structural data significantly [1]. At the moment, only 398 nonredundant membrane protein structures are available by protein structure databases, such as the Protein Data Bank (PDB) or the Protein Data Bank of Transmembrane Proteins [2, 3]. Hence, little is known about membrane proteins. To investigate membrane protein structure and misfolding, other approaches, such as small-molecular-force spectroscopy, have been applied and developed [4]. Mutation-induced membrane protein structure misfoldings are causes of many human diseases, that is, *diabetes insipidus*, *hereditary deafness*, *retinitis pigmentosa*, *cystic fibrosis*, *familial hypercholesterolaemia,* and so on [4–7].

*Diabetes insipidus* (DI) is characterized by polyuria (a daily output of 15–20 L of highly dilute urine) and compensatory polydypsia. In the general population, it is assessed on one case per 25,000–30,000 people [8–10]. Symptoms of DI in newborn infants are irritability, poor feeding, poor weight gain, and dehydration. DI can be differentiated in two classes. First, *central diabetes insipidus* (CDI) is caused by central defects, in which no or an insufficient amount of functional arginine vasopressin (AVP) is released from the pituitary. In contrary, defects in the kidney could cause *nephrogenic diabetes insipidus* (NDI). Four different types of NDI concerning causes and inheritance are known [11–14]:

- acquired NDI, it can originate as a side effect of long, surpassing drug taking (i.e., lithium);
- autosomal recessive inheritable NDI, caused by mutations in AQP2 gene which encodes aquaporin-2;
- dominant inheritable NDI, caused by mutations in AQP2 gene which encodes aquaporin-2;
- X-linked inheritable NDI, caused by mutations in AVPR2 gene which encodes the AVP type-2 receptor (V2R).

The X-linked inheritable variant of NDI is a disorder in which a person affected is unable to concentrate urine in the kidney despite the presence of AVP. This nanopeptide (10 amino acids) acts as an antidiuretic hormone. It binds to V2R as an agonist and induces a cAMP-driven cascade which, as one result, leads to the insertion of aquaporin-2 in the apical collecting duct membrane. As an *α*-helical membrane water channel, the fusion of aquaporin-2 with the cell membrane increases the permeability of apical plasma membranes to water. Thus, water can pass through the apical membrane and leads to the prourine concentration equilibrium. Misfolded V2R mutants trapped in the endoplasmatic reticulum are the main cause for the origin of the X-linked NDI variant. Usually V2R fuses with the basolateral membrane where it is able to bind to AVP. Furthermore, inserted mutants are usually not able to bind with AVP. Thus, trapped V2R mutants or normally inserted but not-functional V2R mutations anticipate the induction of aquaporin-2 insertion which results to polyuria and diuresis. Autosomal recessive or dominant inherited mutations in the AQP2 gene lead to the misfolding of aquaporin-2 and, hence, the insertion of functional aquaporin-2 water channels. This results in the typical NDI symptoms elucidated above as well [15, 16]. Mutations in V2R and aquaporin-2 cause structural instabilities. The analysis of these instabilities plays an important part concerning the understanding process of membrane protein mutation-induced diseases, especially in *diabetes insipidus*.

In this paper, we demonstrate a novel approach for membrane protein stability analysis based on protein energy profiles. The concept of protein energy profiles is a novel coarse-grained model for transforming structural and chemical protein properties to one-dimensional energy representations. A protein energy profile can be calculated by any given protein structure within less than half a second making it valuable for the fast analysis of structure-function relationships. This approach is explained closer in the Material and Methods section. Its application to a structure model of V2R in bound and unbound state to AVP will be elucidated in detail. Additionally, the energy profile based membrane protein analysis is applied to the structure model of the wild-type of aquaporin-2 and selected mutant structure models. Finally, significant differences in the energy characteristics and correlations to experimental data will be discussed in detail.

## 2. Materials and Methods

### 2.1. Description of the Investigated Proteins

#### 2.1.1. V2 Vasopressin Receptor (V2R)

The V2 vasopressin receptor belongs to the class A of G-protein coupled receptors. It contains seven membrane spanning helices which are connected by extracellular and intracellular loops, respectively. The binding of V2R to agonist AVP induces the activation of the protein leading to allosteric structural rearrangements [17]. Once V2R is activated, it is able to interact with the cytosolic G-protein activating adenyl cyclase which triggers a cAMP-driven cascade. As a result aquaporin-2 is inserted in the apical membrane [15, 16]. The binding site of AVP in V2R is located within transmembrane helices II-IV, where the residues 88–96, 119–127, 284–291, and 311–317 are mainly involved in binding [18, 19].

Since there is no known protein structure of V2R, a three-dimensional structure model of V2R was produced using the I-TASSER protein structure modeling pipeline [20]. Basically I-TASSER builds protein models using iterative assembling procedures and multiple threading alignments based on template structures. In Table 1, the PDB_IDs, corresponding biological descriptions and sequence identities to V2R of the employed template structures, are given. Gradient minimization of the modeled structure was produced by means of NAMD2 [21]. Further MD simulation was applied to the model by using CHARMM27 force-field [22]. To study the overall model quality and structural stability, additional MD simulations were performed. The average RMSD of the *C*_*α*_-backbone of the structure model was found to be 2.7 Å.

#### 2.1.2. Aquaporin-2

**Figure 1:**
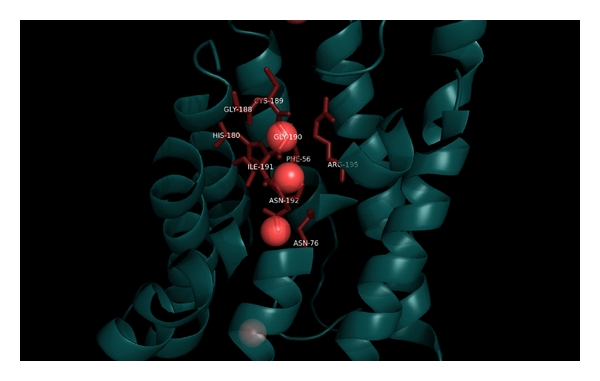
Illustration of the water binding network in aquaporin-1 (PDB_ID 1fqy). Due to high residue conservation, knowledge gained from known aquaporin structures can be assigned to aquaporin-2 reliably. Since there is no high resolution structure of aquaporin-2, this gained knowledge sheds light on the structure-function relationship in aquaporin-2.

Aquaporins provide highly permeable pores for water to cross membranes. Four identical subunits form a stable tetramer spanned through the plasma membrane. Each subunit consists of seven helices which form a pore with 3 Å in diameter. The selectivity for water is achieved mainly by the two asparagine residues 76 and 192 (human aquaporin-1 numbering) [23]. Further selective residues are His180, Gly188, Cys189, Gly190, Ile191, Arg195, Phe56. An illustration of the residues in the aquaporin-1 structure which are involved in water binding is given in Figure 1 [23, 24]. Furthermore, all aquaporins exhibit two highly conserved Asn-ProAla motifs which are located in two opposite meeting *α*-helices in all known aquaporin structures. This indicates the high conservation of this structural feature and its importance in water transport activity. It is shown that these two *α*-helices form a bipolar electric field changing the water molecule orientation and preventing protons to pass the channel. Further molecular simulation studies have revealed a secondary free energy barrier which is induced by Phe56, His180, and Arg195. It is located about 8 Å apart from the primary bipolar electric field at the extracellular side of the protein. As one result of these two bipolar electric fields, a constriction region is formed which allows only a single water molecule to pass the end of the pore. MD simulations of Arg195 mutants revealed the correspondence of the stability of this secondary bipolar electric field to Arg195 and, hence, the influences on water selectivity of the protein [25–27]. Although no high resolution structure of aquaporin-2 is given, all discovered features in known aquaporin structures and knowledge can be assigned reliably to aquaporin-2 due to the mostly high conservation of these residues in all known human aquaporins.

**Figure 2:**
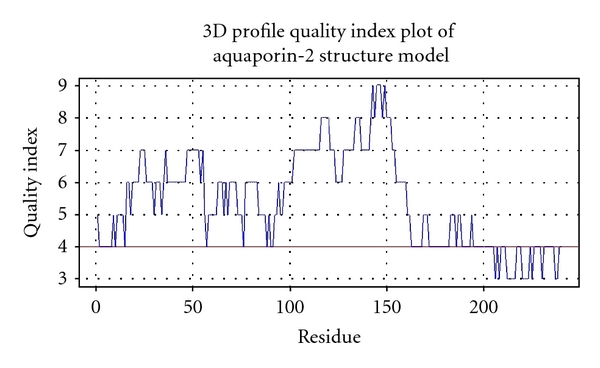
The quality index plot of the aquaporin-2 structure model produced by VADAR. The structure model of aquaporin-2, which is necessary for protein-energy-profile-based analysis, was reevaluated using the structure analysis tool VADAR [29]. One evaluation criterion given by VADAR is the quality index per residue. It indicates weak spots in the model, for example, side chain misfolding, stereochemical overlaps, insufficient atom packing, and so on. Here, the plot points to a high structure model quality.

For the protein-energy-profile-based analysis, a structure model is necessary. Therefore, the most reliable structure model was retrieved from the ModBase database [28]. This model was produced using the high resolution structure of aquaporin-5 as the modeling template (PDB_ID 3d9s). Both proteins share a sequence identity of 68%. The used model exhibits a high coverage of 93% to the template structure. To reevaluate the quality of the model, the protein structure analysis tool VADAR (version 1.8, see [29]) was applied. One evaluation criterion is the quality index which summarizes side chain misfoldings, stereochemical overlaps, and insufficient atom packing for each residue. Quality indexes below 4 are reported as low quality. The quality index plot of aquaporin-2 is shown in Figure 2. As illustrated, the average quality index of all residues of the aquaporin-2 model points to a high quality level with only a few weak spots. Thus, the model can be applied to the protein-energy-profile-based approach.

### 2.2. Theory of Protein Energy Profiles

Since the fundamental work of Anfinsen, which states that the native protein conformation is determined by the sum of amino acid residue interactions and, thus, by the amino acid sequence [30], many coarse- and fine-grained, all atom models describing residue-residue interactions were developed and adapted. They are based either on first principles approaches using physics laws or make use of knowledge of existing experimentally derived structures and statistical analysis. The latter approaches, the so called knowledge-based energy potentials (KBPs), assume that free energy functions describe the behavior of a protein structure and that, according to Boltzmann's principle, the low-energy states are observed with high frequency. KBPs differ in their level of description of system details: ranging from all-atom models and potentials to simplified coarse-grained models. The physics-based approaches to predict protein structure use molecular mechanics force fields which describe proteins at atomic detail and energy terms containing contributions from electrostatic and van der Waals interactions as well as covalent bonding of the polypeptide chain [31, 32]. However, such atomic detail simulations are only feasible for rather small proteins usually shorter than 150 amino acids.

The coarse-grained model for calculating protein energy profiles, which is described here, belongs to the KBPs approaches. Its basis stems from [34–36]. It is similar to the approach described in the work of Eisenberg et al. [37]. Eisenbergs approach transforms the three-dimensional structure of the protein to a one-dimensional representation by analyzing the structural environment of each residue in the structure. This environment is described by the buried surface of the residue side chain and the surface of the side chain which is exposed to polar atoms as well as the local secondary structure of the residue. In contrast, the approach described here analyses the environment of the investigated residue too but approximates its energy by reverting to pseudoenergies derived by statistical physics. These pseudoenergies are based on the tendency of each amino acid for being either buried or exposed to the solvent. Applying the Boltzmann principles to these tendencies, the pseudoenergy of each amino acid can be approximated. For instance, an exposed cysteine holds a higher energy as expected since cysteine is usually buried inside the protein structure [38]. Based on [34, 35], we defined an inside/outside property for generating amino acid buriedness distributions and, hence, allowing the pseudoenergy approximation. Let *i* denote one of the 20 canonical amino acids. *n*_in,*i*_ and *n*_out,*i*_ describe the absolute frequency of the amino acid *i* being assigned as “inside” and “outside” by the inside/outside property, respectively. The inside/outside-property is defined as

(1) $$\begin{array}{cc} & f\left(i\right)\\ & =\{\begin{array}{cc} n_{\text{in},i}++, & ||C_{\alpha ,i}-c||<5\;\text{Å}\vee \left(C_{\alpha ,i}-C_{\beta ,i}\right)\left(C_{\alpha ,i}-c\right)<0\\ n_{\text{out},i}++, & \text{else}, \end{array} \end{array},$$

where *c* denotes the center of mass of all *C*_*α*_ atoms within a 5 Å sphere surrounding *i*. In general, the statistics can be calculated by any given set of proteins but redundancy and physiochemical properties need to be taken into account. For instance, the statistics of *α*-helical membrane proteins differ significantly from statistics derived by globular proteins exclusively. Exchanging statistics or calculating on the basis of a rather inappropriate protein structure set would lead to false conclusions. Here, the statistics were derived by employing this property to 342 nonredundant *α*-helical membrane proteins. The list of these protein structures can be found at the Protein Data Bank of Transmembrane Proteins [1]. Applying the inverse Boltzmann principle, the pseudoenergy *e*_*i*_ of *i* can be approximated as follows:

(2) $$e_{i}=-k_{B}T\ln\left(\frac{n_{\text{in},i}}{n_{\text{out},i}}\right).$$

Since *k*_*B*_ and *T* are declared as constants in this model, both can be omitted from the calculation:

(3) $$\begin{array}{c} e_{i}^{*}=-\ln\left(\frac{n_{\text{in},i}}{n_{\text{out},i}}\right) \end{array}.$$

The energy of the pairwise interactions of *i* to other residues corresponds to the environment of *i* and the environments composition inside the structure [39]. Thus, the expected tendency value *P* of the observed environment composition correlates with the interaction energy of *i*. *P* can be approximated by the derived amino acid distributions:

(4) $$\begin{array}{c} P_{k\in \text{Env}}=\underset{k\in \text{Env}}{\prod }p_{k}=\underset{k\in \text{Env}}{\prod }\left(\frac{n_{\text{in},k}}{n_{\text{out},k}}\right),\\ \ln P_{k\in \text{Env}}=\underset{k\in \text{Env}}{\sum }\ln\left(\frac{n_{\text{in},k}}{n_{\text{out},k}}\right). \end{array}$$

Thus, according to the Boltzmann principle, the energy of the environment *E*_Env_ is defined as

(5) $$\begin{array}{c} E_{\text{Env}}=-\ln P_{k\in \text{Env}} \end{array},$$

and, hence,

(6) $$\begin{array}{c} E_{i}=-\left|\text{Env}\right|\ln\left(\frac{n_{\text{in},i}}{n_{\text{out},i}}\right)-\underset{k\in \text{Env}}{\sum }\ln\left(\frac{n_{\text{in},k}}{n_{\text{out},k}}\right). \end{array}$$

The environment was defined by a contact function *g*(*i*, *j*) adapted from [36] which is denoted as

(7) $$\begin{array}{c} g\left(i,j\right)=\{\begin{array}{cc} 1, & ||C_{\alpha ,i}-C_{\alpha ,j}||\leq 8\;\text{Å,}\\ 0, & \text{else}, \end{array} \end{array}$$

Finally, the total energy of *i* is

(8) $$\begin{array}{c} E_{i}^{*}=\underset{j\in S\setminus i}{\sum }\left[g\left(i,j\right)\left(e_{i}^{*}+e_{j}^{*}\right)\right], \end{array}$$

where *S* defines a given protein structure. By omitting *k*_*B*_ *T* in the model, the resulting *E*_i_* are given in arbitrary unit entities [a.u.] and are direct proportional to energies listed in [*J*] or [kcal mol^−1^]. The protein energy profile of *S* corresponds to the n-tupel of all *E*_i_*.

Similar to the approach discussed in the work of Eisenberg et al. [37], energy profiles can be aligned by means of dynamic programming. Therefore, an energy-energy scoring function was implemented. It is derived by distances between power-equal intervals of the gaussian integral of the energy distribution. For scoring two energies, each energy is assigned to its interval in the gaussian integral. The distance between both integrals corresponds to the pairwise energy score. This scoring is used for aligning two given energy profiles *A* and *B* by dynamic programming, like the Needleman-Wunsch algorithm [40] or the Smith-Waterman algorithm [41]. The estimation of alignment significance is permitted by weighting the resulting score *x*_r_ to the best possible score *x*_opt_ and the average permutation score ${\bar{x}}_{\text{p}}$ which is derived by permuting and realigning the given energy profiles. As discussed in [42], this weighted score is called distance score (dScore) and is defined as

(9) $$\begin{array}{c} \text{dScore}\left(x_{\text{r}}\right)=-\log\left(\frac{x_{\text{r}}-{\bar{x}}_{\text{p}}}{x_{\text{opt}}-{\bar{x}}_{\text{p}}}\right) \end{array}$$

with

(10) $$\begin{array}{c} x_{\text{opt}}\left(A,B\right)=\frac{\delta \left(\left|A\right|+\left|B\right|\right)}{2}. \end{array}$$

**Figure 3:**
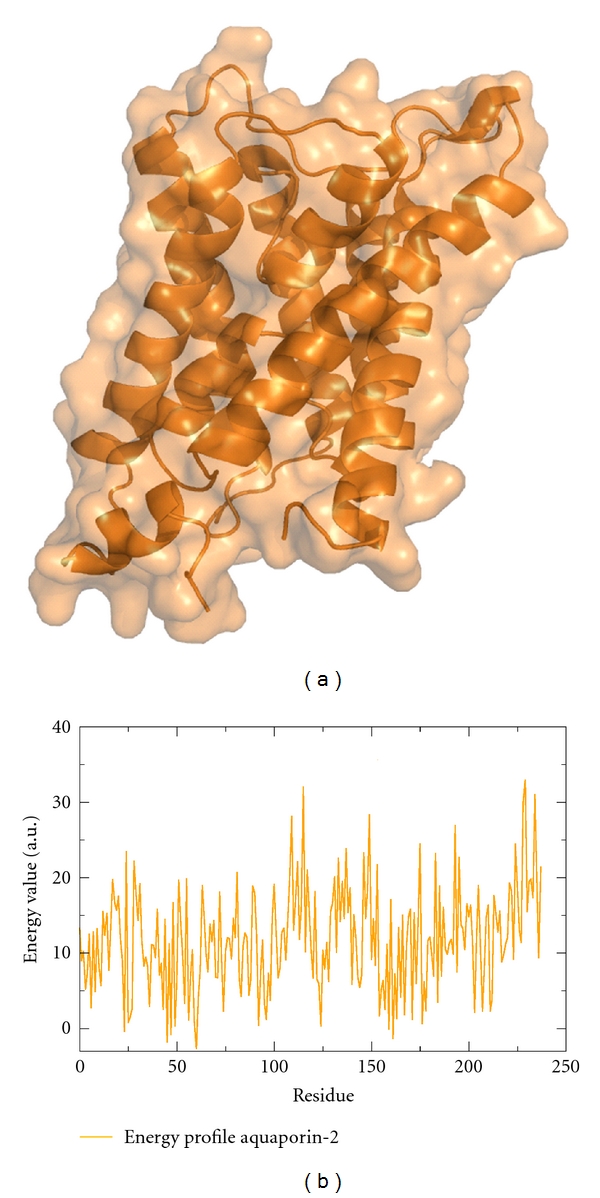
The structure model of aquaporin-2 (a) and its corresponding energy profile (b).

Here, *δ* denotes the best possible pairwise energy score. In general, significant energy profile alignments correspond to dScores of less than 2.5 bans. The alignment of two identical energy profiles corresponds to a dScore of 0 bans (Figure 3).

### 2.3. Correlations of Energy Profiles to Structure and Amino Acid Sequence

The relation of amino acid stability and amino acid energy is explainable by the folding of the protein and its energy landscape. The process of folding can be described as a function of the loss of the free Helmholtz energy within an amino acid interaction energy state. Commonly, a folded protein in its stable state holds the minimized amount of free energy [43]. The energy profile is a transformation of the energy landscape of the protein at the point of minimized free energy. This leads to the conclusion that the energy value of an amino acid *i* given by an energy profile is a transformation of the stability of the amino acid *i* in the structure.

To investigate the correlation of energy profiles to structure and amino acid sequence, seven protein structures (PDB_IDs: 1a1w, 1a3h, 1amm, 1b1j, 1bhe, 1o8w, 3gbs) which share no similarity in structure, sequence, or function were subjected to the PDBeFold service [44] for searching identical and similar protein structures in the Protein Data Bank. Overall, PDBeFold detected 653 significant hits. For each hit, the sequence identity and structure alignment scores (QScores) were saved. Two identical protein structures afford a QScore of 1.0. In contrast, two dissimilar proteins share a QScore of 0.0. Afterwards, the protein energy profiles of the query protein structures and the corresponding hits were calculated and aligned. The Spearman correlation coefficients of the resulting dScores and saved QScores as well as the sequence identities were calculated. The resulting Spearman correlation coefficients are

- QScore to dScore: *ρ*_QScore,  dScore_ = −0.91,
- sequence identity to dScore: *ρ*_seqId,   dScore_ = −0.92,
- sequence identity to QScore: *ρ*_seqId,  QScore_ = 0.94.

**Figure 4:**
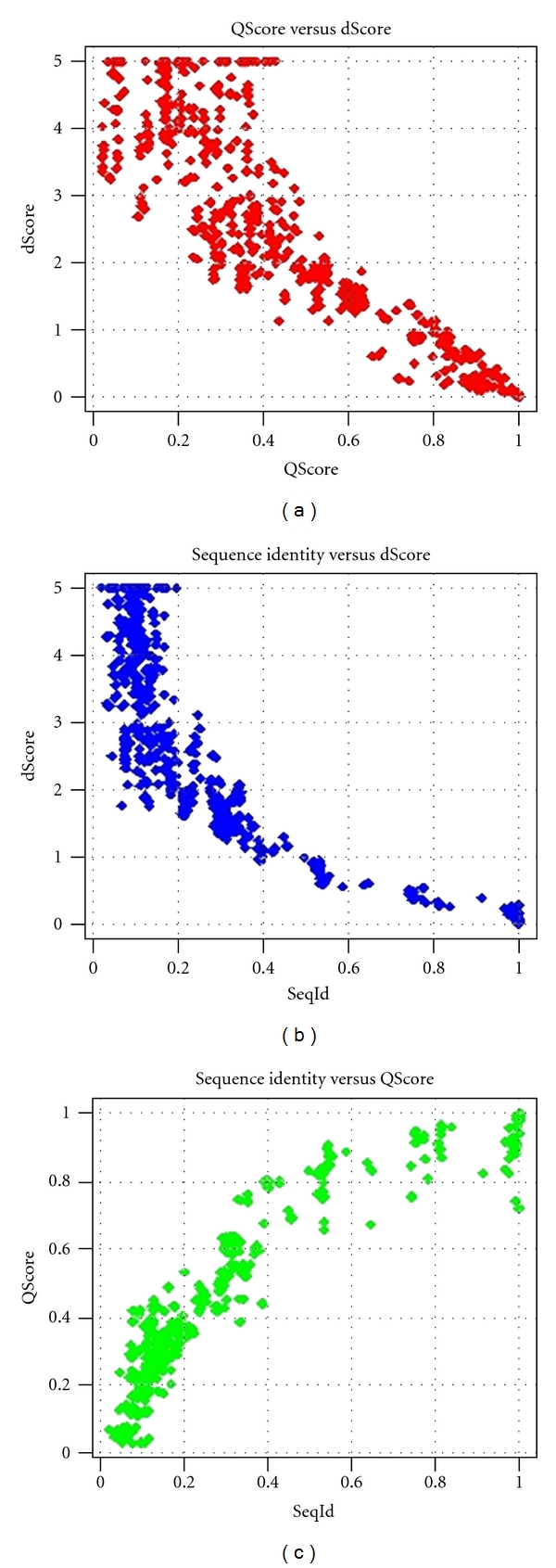
Scatter plots of pairwise energy profile distance scores (dScores), structure alignment scores (QScores), and sequence identities of 7 proteins and their homologs. See text for further information.

The corresponding scatter plots are shown in Figure 4. The high correlation of energy profile similarity to sequence identity and structure similarity leads to the conclusion that energy profile similarity achieves at least the same correlation as sequence identity to protein structure. This implies transitivity of the energy profile of a protein to its amino acid sequence and structure.

Additionally, the correspondence of secondary structure elements to their holding energy was investigated. Therefore, energy profiles of a nonredundant set of 342 *α*-helical membrane proteins were fragmented and labeled according to the secondary structure elements. The fragment length was five residues. 600 fragments were chosen randomly from the entire set of fragments and clustered using neural gas [46]. During the clustering process, the labels were ignored and served only for evaluating the clustering performance. The clustering was evaluated using normalized mutual information (NMI)[47]. This procedure was repeated in 100 iterations to verify the resulting values. Here, the average NMI was found to be at a low level of significance at 0.06 which means that there exists almost no correlation of secondary structures to their holding energy in *α*-helical membrane proteins. The procedure was repeated with labeled fragments according to the membrane topology regions. The NMI was found to be 0.34, which indicates almost linear separability of energy profile fragments according to membrane topology regions. The resulting average NMI of 0.14 of globular protein energy profile fragments labeled according to secondary structure elements shows good clustering. This indicates the correlation of protein energy profiles to structural features of proteins and, thus, the correlation to protein structure stability.

### 2.4. Application of Energy Profiles to V2 Vasopressin Receptor

For investigating the energetics, binding capabilities, and the effect of mutations in the V2 receptor, the structure model of V2R was studied on the level of energy profiles. Therefore, the Molecular Docking Server was used for a docking simulation of the V2R model and the AVP hormone. In [48], it is demonstrated that the semiempirical PM6 partial charges calculation methods, which are implemented in the software of the Molecular Docking server, allowed a docking accuracy of 42 correctly modeled ligand-protein complexes out of a set of 53 ligand-protein complexes determined by X-ray experiments. Regarding the performance of the Molecular Docking Server as well as the energetic trajectories computed while calculating the docking simulation of AVP and V2R (data not shown), the structure of the model and its hormone in bound state was assessed as modeled correctly and used for further analyses. The energy profiles of the V2R model in bound and unbound states were calculated to detect energetic divergences induced by conformational changes during hormone binding.

To detect these binding-induced energetic changes, both derived energy profiles were aligned using a multiple energy profile alignment algorithm (MEPAL) which has been adapted from [42]. In the process, all given protein energy profiles are aligned to each other which results in a distance matrix with the corresponding dScores as matrix entries. In the next step, hierarchical clustering is performed using the unweighted pair-group method with arithmetic mean (UPGMA) and the clustering steps, are recorded. Third, according to the tracked clustering steps all energy profiles are introduced in the progressive multiple energy profile alignment using the same techniques as employed in the pairwise energy profile alignment. Thus, significant divergences and similarities in multiple energy profiles can be detected. Furthermore, this method allows the deduction of consensus energy profiles and energy conservation. The energy at each alignment column in the consensus profile is derived by calculating the pairwise energy scores of all energies at this particular position. The energy with the highest sum of these scores is representing the consensus. The conservation is derived by the sum of the pairwise energy scores and is normalized by the theoretically best possible sum. Hence, the optimal conservation in a column equals 1.0 [49].

### 2.5. Application of Energy Profiles to Aquaporin-2 and Its Homologs

To investigate protein stability in aquaporin-2, a MEPAL of close homolog aquaporins was calculated. The proper proteins are aquaporin-4 (PDB_ID: 3gd8), aquaporin-1 (PDB_ID: 1fqy), the homology model of aquaporin-3 (template PDB_ID: 3ldf), and the structure model of aquaporin-2. The first model was retrieved from the ModBase database and shows less quality than the aquaporin-2 model (data not shown) but is appropriate for further analysis.

### 2.6. Application of Energy Profiles to Aquaporin-2 and Two Well-Described Mutants

For the comparison on the level of energy profiles of aquaporin-2 mutants, two aquaporin-2 models were generated by introducing the two mutations D150E and G196D into the amino acid sequence. Since both mutations are well described in literature [45], correlations of protein energy/stability and experimental observations can be developed.

The modeling of the two mutants was performed by SwissModel [50, 51] using the aquaporin-2 model as template structure. The energy profile of each resulting model was calculated. A MEPAL of the energy profile of the wild-type and the two modeled mutants was generated and investigated for significant differences. Additionally, the distance tree of the energy profiles was computed by means of UPGMA clustering on the basis of the energy profile distance matrix.

## 3. Results and Discussion

The MEPAL output is separated in three parts. The upper part visualizes the energy profile by coloring the residue one letter codes by their energy. The middle section shows the consensus profile. The conservation is illustrated in the lower part.

**Figure 5:**
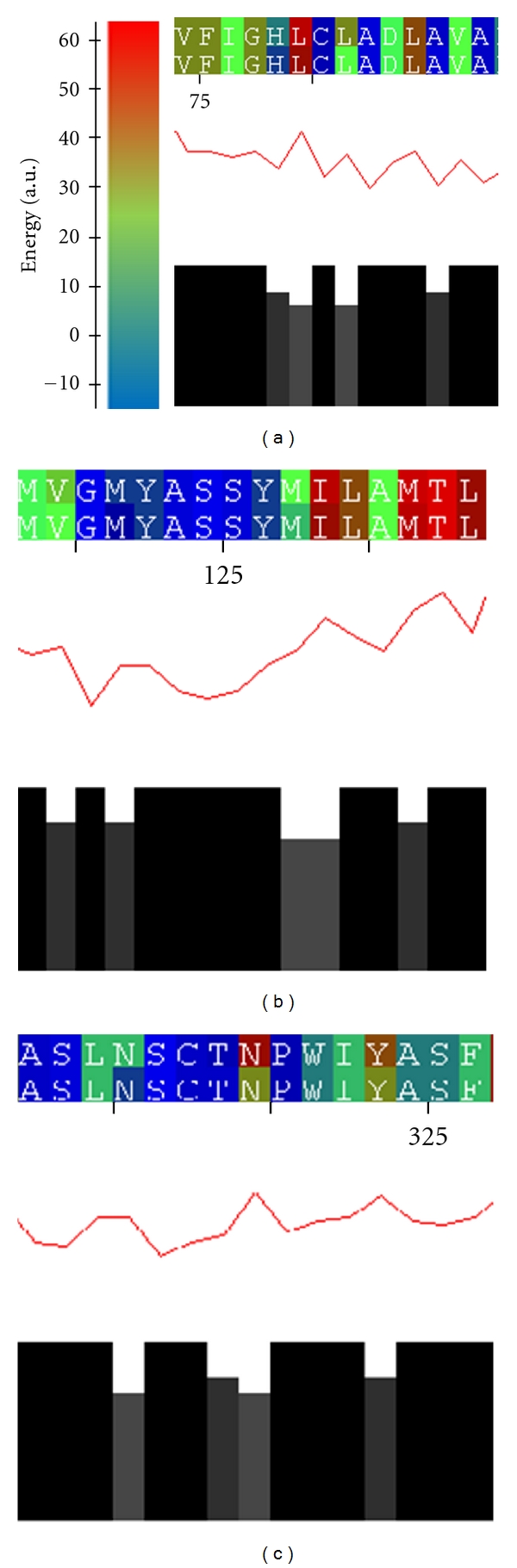
The multiple energy profile alignment (MEPAL) of V2R in bound and unbound state to AVP. Although being overall energetically well conserved, three regions showing distinct energy differences can be detected. These regions correspond to residues involved in AVP binding. This observation points to slight changes and rearrangements in the structure of V2R during the binding process.

The MEPAL of the V2R in bound and unbound state to AVP revealed energetic divergences in the surroundings of the amino acids Ala84 (Figure 5(a)), Ile130 (Figure 5(b)), and Pro322 (Figure 5(c)). This indicates that these amino acids are involved in hormone binding. Their mutations are well described in literature and cause a loss in functionality and hormone affinity [52–56]. This observation emphasizes the quality of the modeled AVP-V2R complex and the coarse-grained energy model discussed in this work. In conclusion, the binding of AVP affects the stability and folding of the structure in only a few spots with rather small structural changes and rearrangements. Hence, this novel energy-profile-based approach brought more evidence and data to the functionality of V2R and the influences of the described mutations.

**Figure 6:**
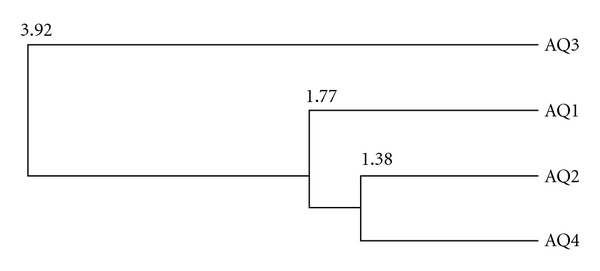
The distance tree computed by the energy profile distance matrix of aquaporin-1, -2, -3 and -4 using the unweighted pair group method with arithmetic mean (UPGMA). The energy profiles of aquaporin-1, -2, and -4 show high similarities. The longer distance of aquaporin-3 correlates with higher differences to the energy profiles of the other aquaporins.

The analysis of the distance tree generated by the energy profile distance matrix of the investigated aquaporins indicates high similarities between the energy profiles of aquaporin-2 and aquaporin-4. The derived energy profile distance of aquaporin-3 to the other structures corresponds to significant but less similarity (see Figure 6).

**Figure 7:**
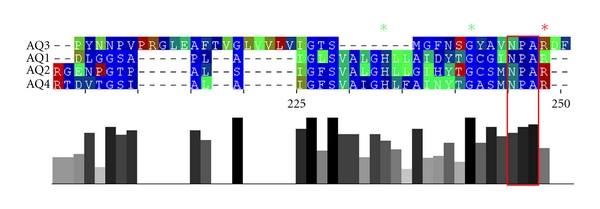
Part of the MEPAL output of aquaporin-1, -2, -3, and -4. Highly sequence conservations are highlighted by red stars. Highlighted by a red box, the second of the two Asn-ProAla motifs can be seen. For detailed discussion, see the text.

The MEPAL alignment of the four aquaporins shows several energetically highly conserved regions. Two of them correspond to the opposite orientated Asn-ProAla motifs. The MEPAL output of the surrounding area of the second Asn-ProAla motif is shown in Figure 7. In this figure, the second Asn-ProAla motif is highlighted by a red box.

The energetic conservation of these motifs and their surrounding amino acids confirms the importance of these residues in water transport. Additionally, the residues Gly188 (highlighted by the right green star in Figure 7), Phe56, Cys189, Ile191, and His180, which are involved in water transport as well, show differences in sequential and energetic conservation (not shown in Figure 7). In more detail, the conserved amino acids Gly188 and Phe56 show slight or no differences at all concerning their energies. Cys189 and Ile191 show no conservation in aquaporin-2, -3, and -4; but these changes have no effect on the level of energy profiles. His180 (highlighted by the left green star in Figure 7) shows sequential and energetic conservation in all aquaporins except aquaporin-3. We postulate that these slight differences do not affect the water flux significantly. A further point of interest lies in Arg195 (highlighted by the red star in Figure 7). This residue is conserved in all four proteins but varies energetically. These differences arise from conformational changes of the residue and the structural environment. Based on the facts we referred to in Section 2.1.2, we postulate that these divergences between aquaporin-1, -2, -3, and -4 lead to a changes in the residue Gly188 and its surrounding residues influencing the transport selectivity and the water flux. It also needs to be said that the significant differences in the energy profile progression between aquaporin-3 and the other structures might result by the less reliable aquaporin-3 model.

**Figure 8:**
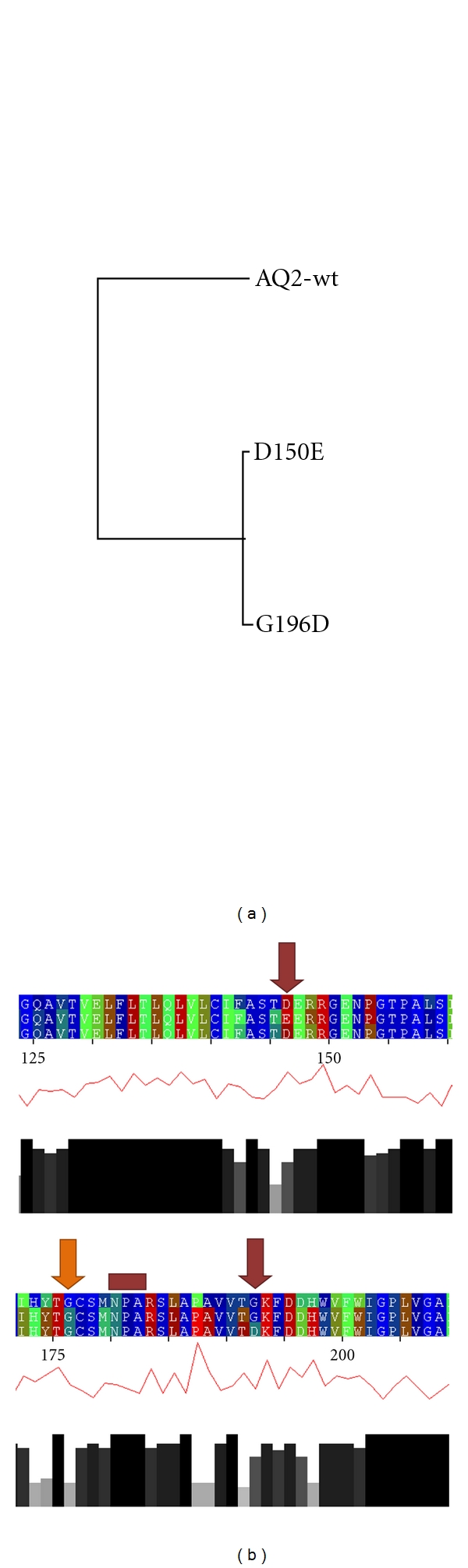
The MEPAL output of the energy profile of the aquaporin-2 wild type and its two mutants D150E and G196D. The distance tree derived by MEPAL indicates the distinct similarity of the energy profiles of the mutants (a). This is visualized by the MEPAL output (b). In the regions, which surround the mutated residues, similarities in energy profile progression can be seen. Thus, it is supported that both mutations affect protein stability and the transcellular water flux as described in literature [45].

The distance tree of the aquaporin-2 wild type and its two modeled mutants (D150E and G196D) indicates strong similarities between the energy profiles of the two mutants (Figure 8(a)). This leads to the conclusion that both mutants induce the same energetic, structural, and functional changes. It needs to be addressed that automated modeling techniques might not be sensitive enough to model single-point-mutated structures based on a template. Two scenarios arise from these concerns. First, two differing single-point-mutated models and the template differ in structure significantly. Or, as the second scenario, both models and the template show identical folds with respect to backbone conformation and side-chain conformations as well. In the first scenario, the resulting three energy profiles would differ significantly from each other leading to a long-branched, stretched distance tree. In the second scenario, the pairwise comparison of all energy profiles would result to a distance tree with very short branches indicating the given high energy profile similarity. But as seen in the distance tree of the aquaporin-2 wild type and its two modeled mutants (Figure 8(a)), the energy profiles of the mutants match very well and differ to the energy profile of the wild type significantly. This is a strong indication that both models can be assessed as modeled correctly.

While both mutations led to energetic variations in the entire energy profiles, we focused our discussion on the mutation sites (Figure 8(b), red arrows). The mutation D150E induces an energetic increase of the two surrounding residues, thus, decreasing the energetic conservation at these positions (Figure 8(b), top). Interestingly, in this region, the mutation G196D induces almost the same energetic increase as D150E. At the mutation site of the modeled G196D variant, the mutation induces only slight energetic divergences in the sequentially surrounding residues (Figure 4(b), bottom). Furthermore, in this region, the G196D mutation leads to the same energetic variations as the D150E mutation. Interestingly, both mutations do not affect the energetic conservation of the Asn-ProAla motif (highlighted by a red rectangle). Additionally, we point to the energetic changes of G188, a residue involved in water transport (highlighted by an orange arrow). Both mutations lead to an energetic increase of Gly188 and reduce the energetic conservation in these three investigated energy profiles. Thus, it supports the findings that the mutations D150E and G196D affect the transcellular water transport as described in literature [45].

## 4. Conclusion

 We investigated the stability of membrane proteins involved in NDI on the basis of theoretical assumptions. These theoretical methods are based on the so-called energy profile calculation which is demonstrated in this work. On the basis of these stability analyses, we were able to enforce evidence for water flux reduction induced by well-described mutations of aquaporin-2. Furthermore, the correlation of residue and energetic conservation of amino acids involved in water transport was detected. Additionally, we focused on selected point mutations in V2R and their influences in hormone affinity. Based on our data, we were able to enforce evidence described in literature. This indicates that our approach can be successfully employed in the study of other disease-linked membrane protein mutations. Especially, conserved energy profile regions were identified. In general, this approach has proved its applicability regarding similar biological questions.

## Figures and Tables

**Table 1:** Overview of the used template structures applied in modeling the V2R receptor structure.

PDB_ID	Description	Sequence identity to template [%]
2ks9	Substance-P receptor	19
2rh1	B2-adrenergic G-protein coupled receptor	22
1l9h	Bovine rhodopsin	18
